# Methylation and transcription patterns are distinct in IDH mutant gliomas compared to other IDH mutant cancers

**DOI:** 10.1038/s41598-019-45346-1

**Published:** 2019-06-20

**Authors:** Dusten Unruh, Makda Zewde, Adam Buss, Michael R. Drumm, Anh N. Tran, Denise M. Scholtens, Craig Horbinski

**Affiliations:** 1 Department of Neurological Surgery, Northwestern University, Chicago, IL 60611 USA; 2 Department of Preventive Medicine, Northwestern University, Chicago, IL 60611 USA; 3 Department of Pathology, Northwestern University, Chicago, IL 60611 USA

**Keywords:** CNS cancer, CNS cancer

## Abstract

Mutations in *isocitrate dehydrogenases* 1 and 2 (IDH^mut^) are present in a variety of cancers, including glioma, acute myeloid leukemia (AML), melanoma, and cholangiocarcinoma. These mutations promote hypermethylation, yet it is only a favorable prognostic marker in glioma, for reasons that are unclear. We hypothesized that the patterns of DNA methylation, and transcriptome profiles, would vary among IDH^mut^ cancers, especially gliomas. Using Illumina 450K and RNA-Seq data from The Cancer Genome Atlas, we show that of 365,092 analyzed CpG sites, 70,591 (19%) were hypermethylated in IDH^mut^ gliomas compared to wild-type (IDH^wt^) gliomas, and only 3%, 2%, and 4% of CpG sites were hypermethylated in IDH^mut^ AML, melanoma, and cholangiocarcinoma, relative to each of their IDH^wt^ counterparts. Transcriptome differences showed pro-malignant genes that appear to be unique to IDH^mut^ gliomas. However, genes involved in differentiation and immune response were suppressed in all IDH^mut^ cancers. Additionally, IDH^mut^ caused a greater degree of hypermethylation in undifferentiated neural progenitor cells than in mature astrocytes. These data suggest that the extent and targets of IDH^mut^-induced genomic hypermethylation vary greatly according to the cellular context and may help explain why IDH^mut^ is only a favorable prognostic marker in gliomas.

## Introduction

Epigenetic modifications control gene expression via mechanisms that are highly coordinated throughout the life of a cell. In addition to regulating the development of distinct cell lineages, epigenetic modifications can also promote malignant transformation and cancer progression^1^. Methylation of a CpG site within or near a gene can change its expression, usually by suppressing it. If this happens to a tumor-suppressor gene, oncogenesis may occur^2^.

The mechanisms by which aberrant methylation occurs, and its consequences in cancer, are becoming better understood. One such mechanism involves point mutations in isocitrate dehydrogenases 1 and 2 (collectively “IDH^mut^”). These metabolic enzymes normally convert isocitrate into α-ketoglutarate, but mutations in key arginine residues that normally bind isocitrate substrate cause a radical change in enzymatic activity, wherein mutant enzyme converts α-ketoglutarate into D-2-hydroxyglutarate (D2HG)^3^. D2HG then acts as a competitive inhibitor of other enzymes that require α-ketoglutarate as a cofactor, including certain DNA demethylases, leading to genomic CpG hypermethylation and globally altered transcription^4,5^. IDH^mut^-induced hypermethylation may lead to suppression of cellular differentiation, thereby prolonging the window in which additional oncogenic “hits” can occur^6^.

IDH^mut^ has been identified in a variety of malignancies, but the most common include infiltrative glioma^7^, acute myeloid leukemia (AML)^8^, melanoma^9^, cholangiocarcinoma^10^, and cartilaginous tumors^11^. Even though IDH^mut^ has the same basic effects on the biochemistry and methylome of every cancer in which it has been detected, glioma is the only cancer in which IDH^mut^ is a favorable prognostic marker^6,12–17^. The reasons for this are still unclear. In our recently published work, we found that the methylation-dependent suppression of Tissue Factor (TF) contributes to the reduced thrombogenicity and reduced malignancy of IDH^mut^ gliomas, and that methylation and suppression of the gene encoding TF, *F3*, is far greater in IDH^mut^ gliomas than in other IDH^mut^ cancers^18,19^. This prompted a broader comparative analysis of the major IDH^mut^ cancers in The Cancer Genome Atlas, demonstrating great diversity in DNA methylation patterns, resultant transcriptomic profiles, and specific genes and biological pathways altered by IDH^mut^ depending on cellular context.

## Results

### Patient cohort characteristics

**Figure 1:**
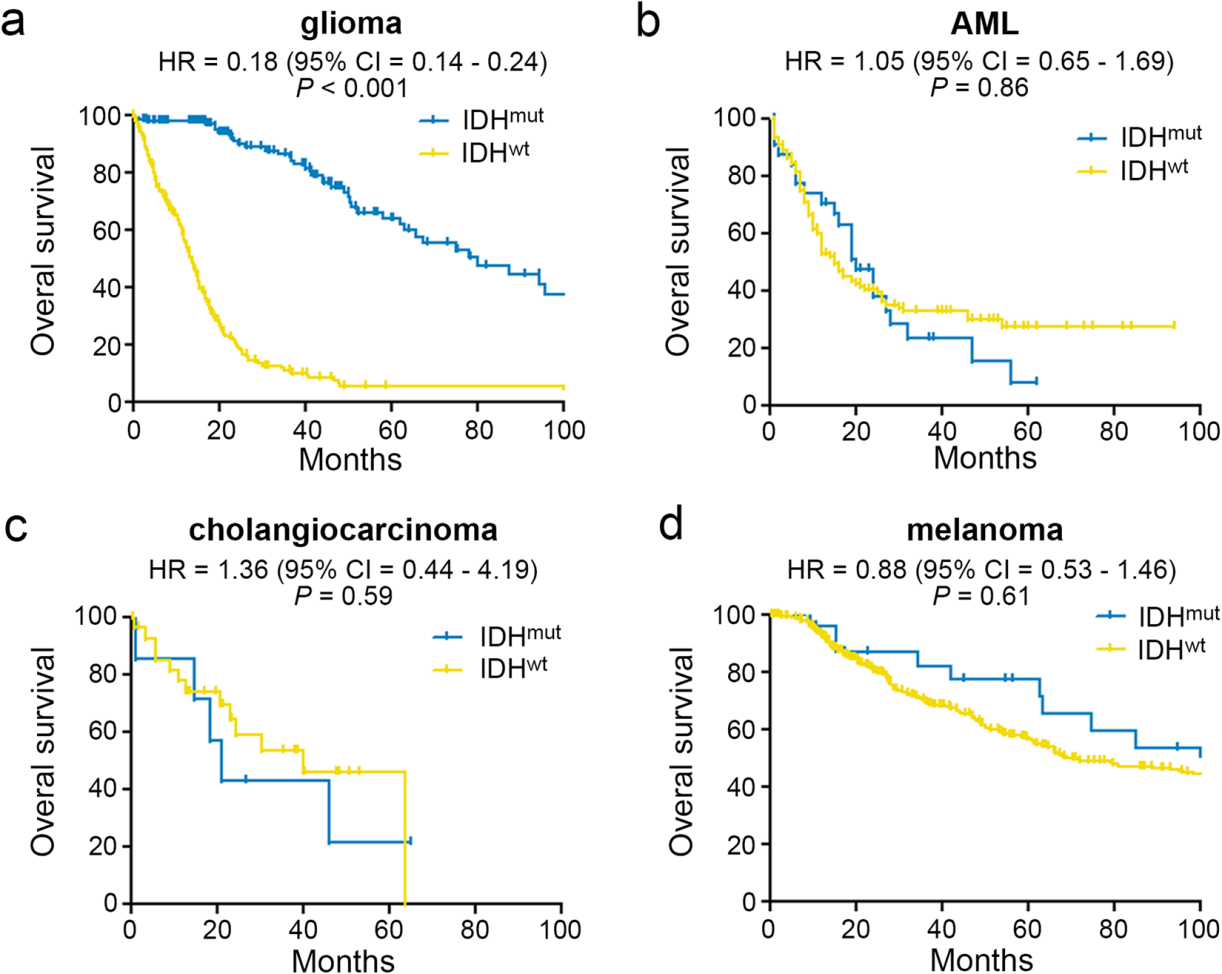
Overall survival of cancer patients stratified according to IDH mutation status. Kaplan-Meier curves of overall survival in IDH^wt^ and IDH^mut^ (**a**) glioma (IDH^mut^ N = 151; IDH^wt^ N = 302), (**b**) AML (IDH^mut^ N = 34; IDH^wt^ N = 137), (**c**) cholangiocarcinoma (IDH^mut^ N = 7; IDH^wt^ N = 29), (**d**) and melanoma (IDH^mut^ N = 25; IDH^wt^ N = 439).

Whole-genome DNA methylation profiles of glioma, AML, cholangiocarcinoma, and melanoma from The Cancer Genome Atlas (TCGA) were grouped by isocitrate dehydrogenase 1 and 2 mutation status, referred to as IDH^mut^ or IDH^wt^. The glioma cohort included 647 patients that consisted of World Health Organization (WHO) grade II-IV gliomas (427 IDH^mut^ and 220 IDH^wt^). The AML cohort included 194 patients (15 IDH^mut^ and 179 IDH^wt^), TCGA had 45 cholangiocarcinomas (7 IDH^mut^ and 38 IDH^wt^), and melanomas consisted of 475 cases (23 IDH^mut^ and 452 IDH^wt^). Chondrosarcomas are not represented in TCGA. Clinical data, including age, gender, race and ethnicity are summarized in Table 1. Survival plots are depicted in Fig. 1. IDH^mut^ only had a significant favorable survival advantage in glioma patients (median 79.9 versus 13.3 months, HR = 0.18, 95% CI = 0.14–0.24, *P* < 0.0001), and was not associated with overall survival in the available AML, cholangiocarcinoma, or melanoma data (Fig. 1).

**Table 1:** Clinical data summaries for TCGA cohorts.

	Glioma n = 647	AML n = 194	Cholangio n = 36	Melanoma n = 472
**IDH status – N (%)**				
Mutant	427 (66.0)	15 (7.7)	7 (19.4)	23 (4.9)
Wildtype	220 (34.0)	179 (92.3)	29 (80.6)	449 (95.1)
**Gender – N (%)**				
Female	284 (44.1)	89 (45.9)	20 (55.6)	180 (38.1)
Male	360 (55.9)	105 (54.1)	16 (44.4)	292 (61.9)
**Race - N (%)**				
American Indian/Alaska native	1 (0.1)	0 (0.0)	0 (0.0)	0 (0.0)
Asian	8 (1.3)	2 (1.0)	3 (8.3)	12 (3.6)
Black or African American	44 (7.0)	13 (6.8)	2 (5.6)	1 (0.2)
White	575 (91.6)	177 (92.2)	31 (86.1)	449 (97.2)
**Ethnicity – N (%)**				
Hispanic/Latino	32 (5.6)	1 (0.5)	2 (5.7)	11 (2.4)
Not Hispanic/Latino	539 (94.4)	190 (99.5)	33 (94.3)	448 (97.6)
Age in years; mean (SD)	47.0 (14.9)	58.0 (16.0)	63.5 (12.8)	58.7 (15.7)

Cholangio = cholangiocarcinoma.

### Patterns of genomic methylation vary across IDHmut cancers

**Figure 2:**
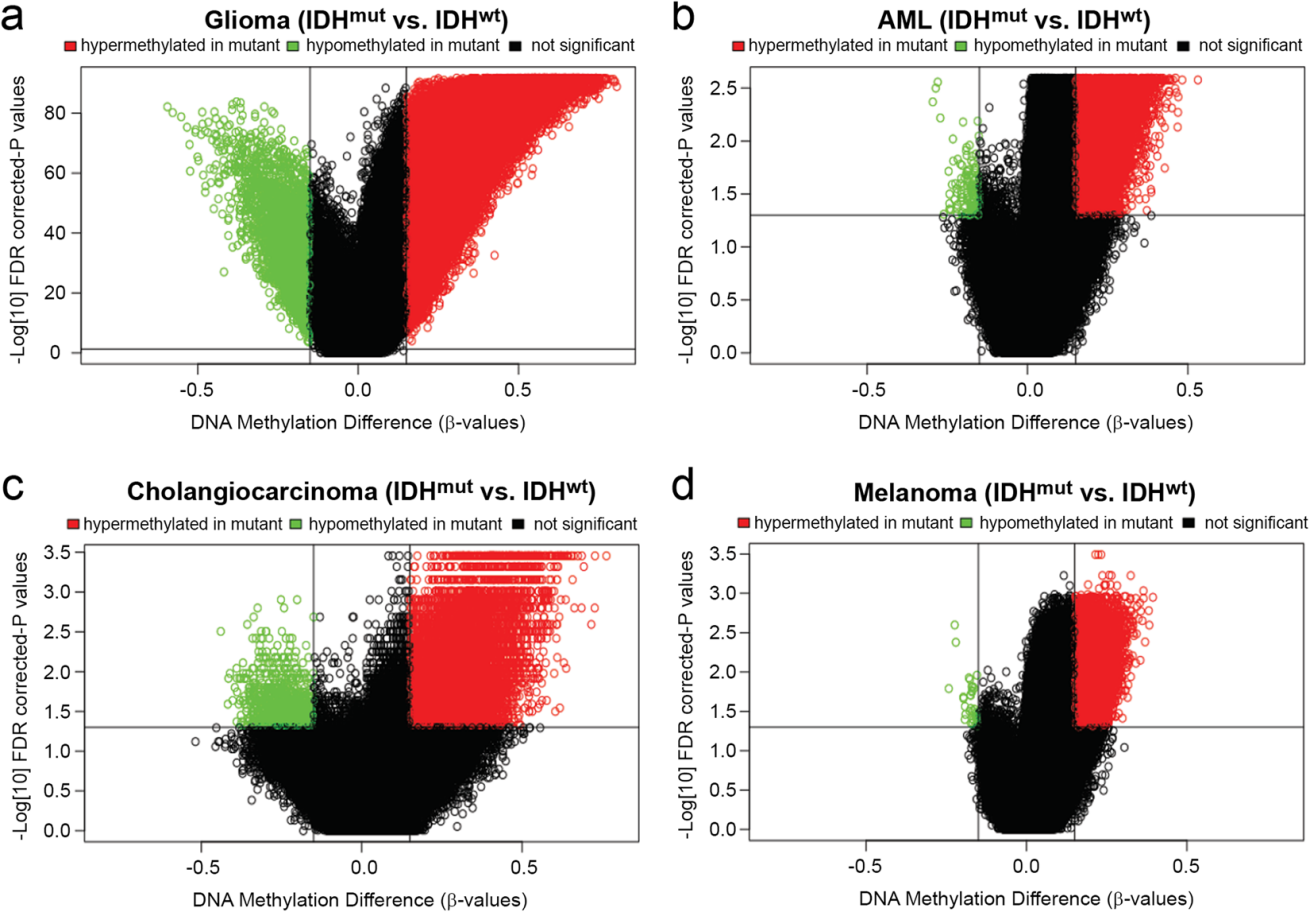
Methylation levels across IDH^mut^ cancers. Volcano plots comparing CpG methylation levels of IDH^mut^ versus IDH^wt^ in (**a**) glioma, (**b**) AML, (**c**) cholangiocarcinoma, and (**d**) melanoma. Difference of mean methylation (*x*-axis) and significance of the difference (*y*-axis). Each point represents a unique CpG site. Hypermethylation is represented by a delta beta ≥0.15 and FDR-corrected *P*  < 0.05 shown in red, and hypomethylation by delta beta ≤−0.15 and FDR-corrected *P*  < 0.05.

To determine the methylation profile for each cohort, β-values for CpG sites that met mapping quality thresholds in the TCGA DNA Methylation Liftover Pipeline^20^ for all samples within a cancer type were analyzed, yielding 365,092 CpG sites for glioma, 393,152 CpG sites for AML, 379,101 CpG sites for cholangiocarcinoma, and 373,827 CpG sites for melanoma. A CpG site with a mean delta β-value > 0.15 (<0.15) for IDH^mut^ relative to the matching IDH^wt^ cancer, with FDR *P* < 0.05, was considered to be hypermethylated (hypomethylated). IDH^mut^ was associated with genomic hypermethylation among all cancers, yet hypomethylation was also observed (Fig. 2). Gliomas showed extensive hypermethylation; of the 365,092 analyzed CpG sites, 70,591 (19%) were hypermethylated in IDH^mut^ glioma compared to IDH^wt^ gliomas, whereas the others ranged between 2–4% (Supplementary Table S1). To rule out the possibility that IDH1^wt^ gliomas are just abnormally hypomethylated relative to other IDH1^wt^ malignancies, a similar analysis was done directly comparing IDH^mut^ gliomas to the other IDH^mut^ cancers. In this setting, IDH^mut^ gliomas showed much greater levels of hypermethylation when compared with other IDH^mut^ cancers (Supplementary Table S2). The number of CpG sites hypermethylated in the other cancers were 13,128 (3%) in AML, 11,763 (3%) in cholangiocarcinoma, and 8,663 (2%) in melanoma relative to each of their IDH^wt^ counterparts (Fig. 2b–d). While smaller sample sizes may have contributed to an overall lower frequency of statistically significant findings for AML, cholangiocarcinoma, and melanoma, differences in β-values for IDH^mut^ compared to IDH^wt^ tended to be more extreme across CpG sites for gliomas than for the other cancers, regardless of *P*-value. Differences in β-values approached 0.8 at the extremes for gliomas, while observed differences in β-values for the other cancers tended to be 0.5 or less (Fig. 2). Interestingly, IDH^mut^ gliomas also showed the greatest degree of hypomethylation when compared with IDH^mut^ AML, cholangiocarcinoma, and melanoma (Fig. 2). Overall, these results suggest that the methylation profiles associated with IDH^mut^ vary greatly according to cellular and tissue contexts.

**Figure 3:**
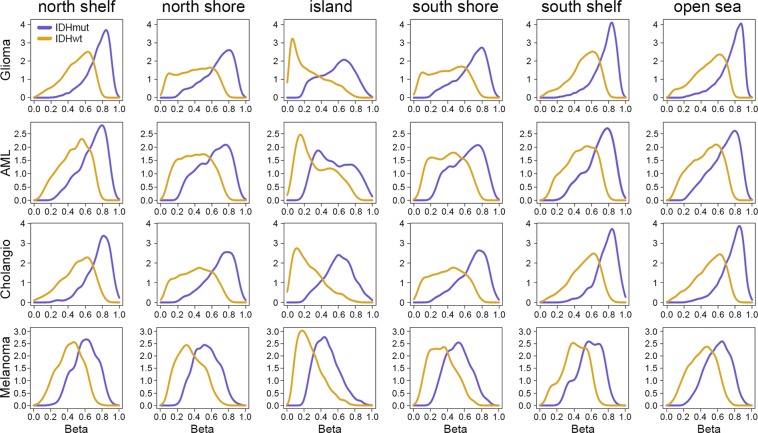
Distribution of mean site-specific CpG methylation levels by genomic region for CpG sites identified as hypermethylated in IDH^mut^ versus IDH^wt^ tumors. The density of probes at each CpG site (*y*-axis) is plotted versus methylation β-values (*x*-axis). Islands are genomic areas with relatively high CpG content flanked by shores (up to 2 kilobases) and shelves (2–4 kilobases from island). North shore and shelf represent 5′ end, and south shore and shelf represent 3′ end. The open sea represents the rest of the genome. Gold lines represent CpG methylation in IDH^wt^ tumors and blue lines represent CpG methylation in IDH^mut^ tumors.

To further investigate the unique methylation profiles observed among IDH^mut^ TCGA cancers, methylation sites were grouped into six genomic regions relative to CpG islands: (1) north shelf, (2) north shore, (3) island, (4) south shore, (5) south shelf, (6) open sea^21^. ‘Shore’ regions immediately flank each CpG island up to 2 kilobases away, ‘shelf’ regions extend outwards from the ‘shore’ up to 2 kilobases away from the shelf, and ‘open sea’ indicates the rest of the genome. The ‘north’ region represents the 5′ end, and ‘south’ the 3′ end. Within each cancer type, IDH^mut^ tumors consistently showed increased methylation across all genomic regions relative to their IDH^wt^ counterparts (Fig. 3). However, the most striking IDH^mut^-IDH^wt^ difference in methylation among cancer types was found in CpG islands, wherein IDH^mut^ gliomas showed a greater increase in CpG island methylation, relative to IDH^wt^ gliomas, than was found in other IDH^mut^ cancers. IDH^mut^ gliomas also showed increased methylation across all genomic regions when directly compared with other IDH^mut^ cancers (Supplementary Fig. S2). This suggests that, while IDH^mut^ is associated with increased CpG methylation across all major genomic regions, the greatest differences occur within CpG islands.

### Methylome and transcriptome changes in IDH^mut^ gliomas are distinct from other IDH^mut^ cancers

**Figure 4:**
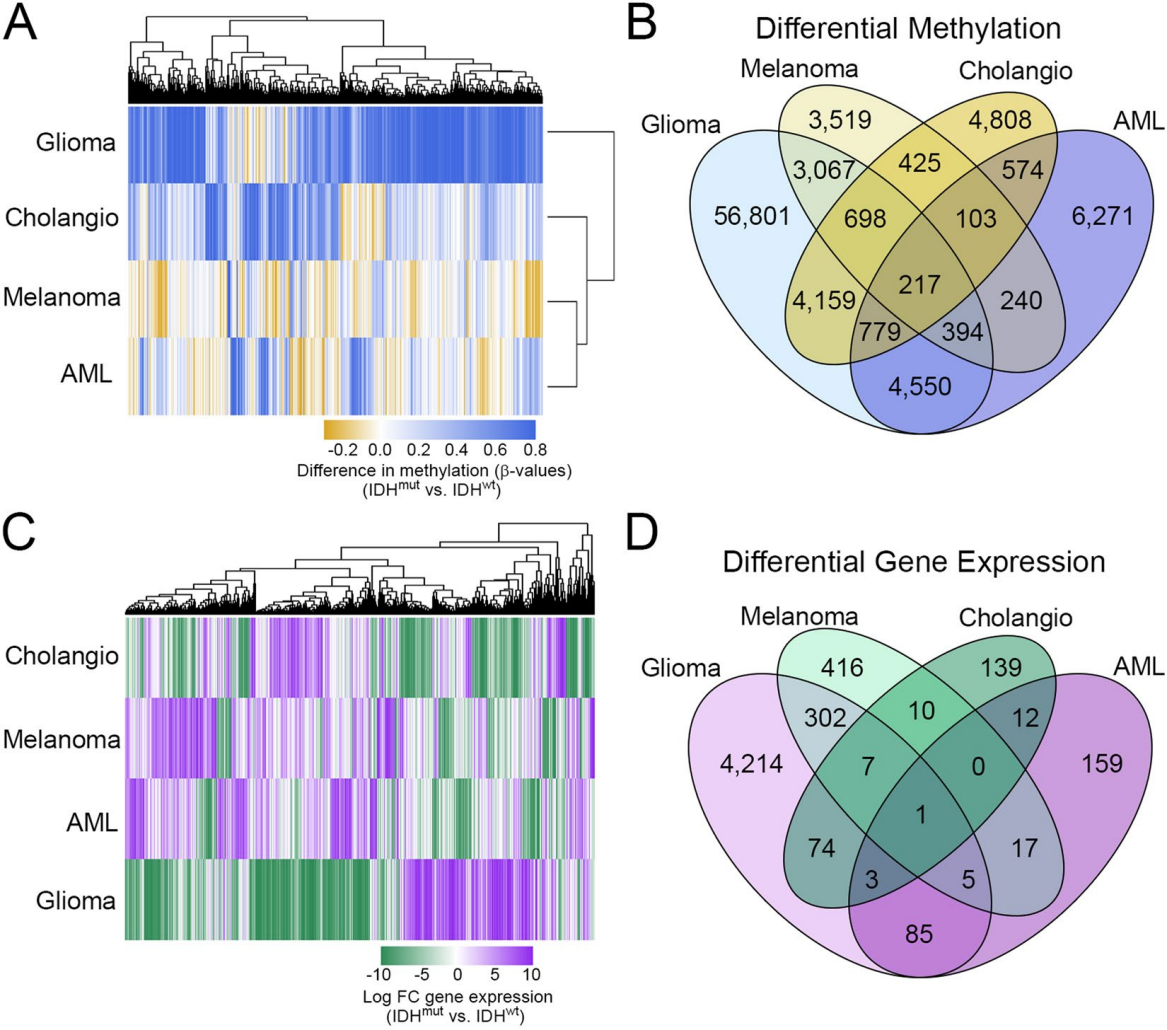
Differential methylation and transcription signatures between IDH^mut^ and IDH^wt^ groups within each cancer type. (**A**) Methylation heatmap of differences in CpG site beta values between IDH^mut^ and IDH^wt^ tumors for all genes demonstrating statistically significant hypermethylation in at least one tumor type. (**B**) Venn diagram representing the overlap of hypermethylated genes of IDH^mut^ cancers compared with IDH^wt^ control. (**C**) Gene expression heatmap of log fold change (FC) gene expression between IDH^mut^ and IDH^wt^ tumors for all genes demonstrating statistically significant differential expression in at least one tumor type. (**D**) Venn diagram representing the overlap of differentially expressed genes of IDH^mut^ cancers compared with IDH^wt^ control.

Hierarchical cluster analysis of differences in β-values for genes that were hypermethylated in IDH^mut^ compared to IDH^wt^ cancers, in at least one cancer type, highlighted the distinct differential methylation profile for CpG sites between IDH^mut^ and IDH^wt^ subsets across tumor types. In particular, many CpG sites with substantial hypermethylation in gliomas (dark blue) consistently showed far more modest differences in methylation for IDH^mut^ versus IDH^wt^ (yellow) in the other tumors, regardless of statistical significance (Fig. 4A). Of these CpG sites meeting formal criteria for differential methylation, 56,801 were unique to glioma, and were not identified as differentially methylated in any other IDH^mut^-IDH^wt^ tumor pairings. Notably, despite smaller sample sizes for AML, cholangiocarcinoma, and melanoma, statistical analyses did identify unique sites of differential methylation in those tumors (6,271 AML; 4,808 cholangiocarcinoma; 3,519 melanoma). Since those CpG sites were not significantly methylated in IDH^mut^ gliomas, even though the glioma cohort was much larger, these results indicate that a great deal of IDH^mut^-related CpG site methylation is tissue-specific. Only 217 similar CpG sites were hypermethylated across all IDH^mut^ cancers, compared to all IDH^wt^ cancers (Fig. 4B). Transcriptome comparisons showed similar results, with IDH^mut^ gliomas showing unique alteration of more mRNA transcripts (4,214) than any other tumor type (Fig. 4C,D). Stratifying IDH^mut^ gliomas into oligodendroglioma and astrocytoma revealed a large amount of similar CpG sites hypermethylated when compared to IDH^wt^ gliomas (Supplemental Fig. S1a,b). There were some variances in differential gene expression between IDH^mut^ oligodendrogliomas and astrocytomas. Oligodendrogliomas showed unique down regulation of genes linked to angiogenesis, cell proliferation, and integrin binding (Supplemental Fig. S1c). Hierarchical clustering of methylation sites again demonstrated that, whereas the methylation patterns of IDH^mut^ AML, cholangiocarcinomas, and melanomas were relatively similar, IDH^mut^ gliomas were quite distinct (Fig. 4A). Glioma showed the greatest IDH^mut^-IDH^wt^ differential gene expression (4,214), compared to AML (159), cholangiocarcinoma (139), and melanoma (416) (Fig. 4D). IDH^mut^ gliomas also had the most concordance of genes that were both hypermethylated and differentially expressed when compared with other cancers (Supplementary Table S3).

**Figure 5:**
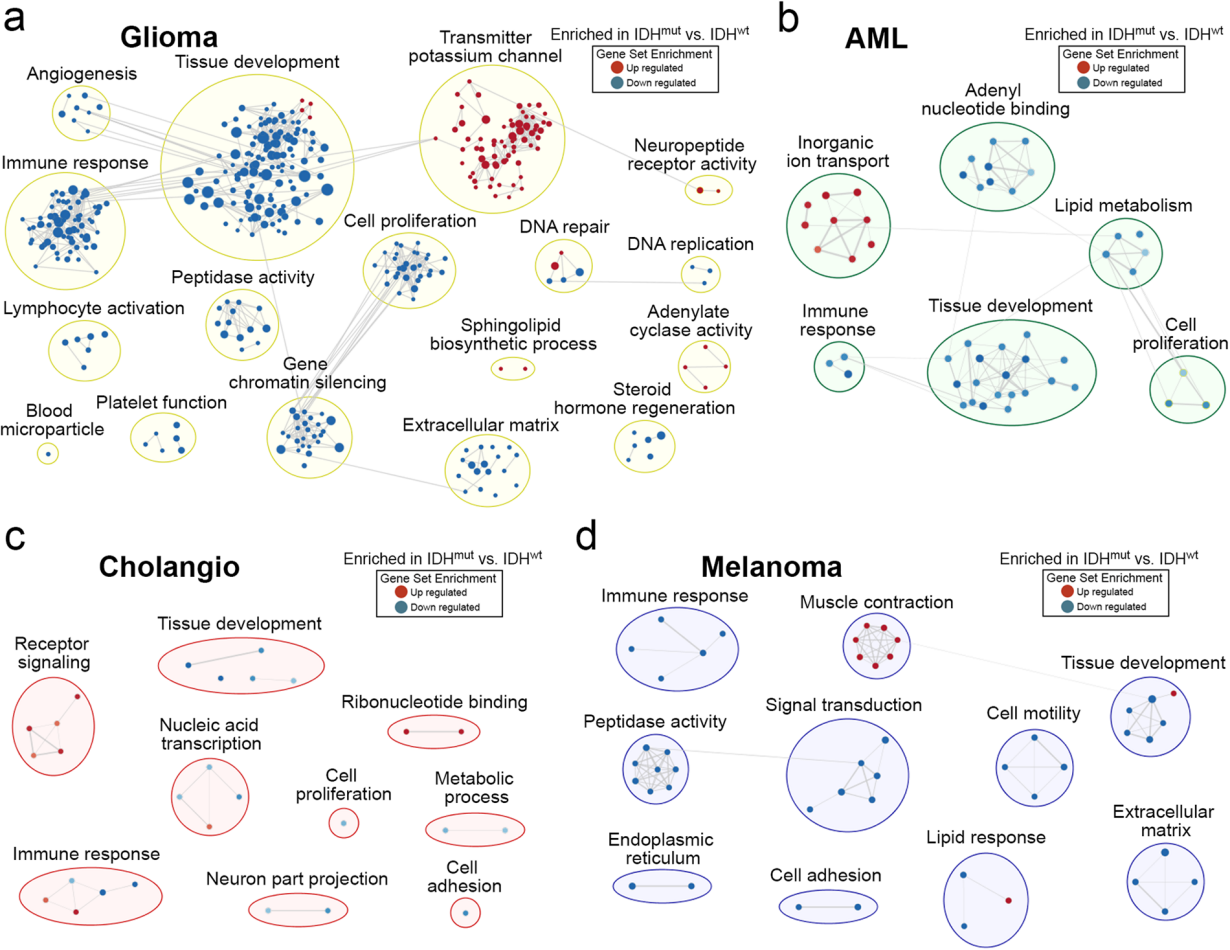
Gene Set Enrichment Analysis (GSEA) showing gene sets in an interaction network for (**a**) glioma, (**b**) AML, (**c**) cholangiocarcinoma, and (**d**) melanoma. Each circular node represents enriched gene sets, where node color intensity corresponds with statistical significance (*P*-value). Blue nodes represent negative enrichment and red nodes represent positive enrichment. Edges (grey connection lines) represent overlap between gene sets with line thickness correlating to the degree of overlap. Networks of nodes that reflect generic function were circled and assigned group labels.

To better understand how these methylation and transcription differences among IDH^mut^ cancers might impact cell biology, we performed gene set enrichment analyses (GSEA) of genes that were differentially expressed within each pair of IDH^mut^ and IDH^wt^ cancers. Using an FDR-corrected *P* < 0.05, we found the following differentially expressed genes: 4,691 (glioma); 282 (AML); 246 (cholangiocarcinoma); 758 (melanoma). Compared to IDH^wt^ gliomas, IDH^mut^ gliomas showed increased expression of 1,629 genes and decreased expression of 3,063 genes (Supplementary Spreadsheet). Glioma GSEA of IDH^mut^ and IDH^wt^ tumors revealed a down-regulation of multiple biological processes; most notable were tissue development (GO:0009888), immune response (GO:0006955), angiogenesis (GO:0001525), and cell proliferation (GO:0008283) (Fig. 5a). GSEA of AML, melanoma, and cholangiocarcinoma showed enrichment of fewer pathways (Fig. 5b–d), which is likely related to smaller sample sizes and lower statistical power. However, all IDH^mut^ cancers showed suppression of GSEA pathways involved in tissue development (GO:0009888) and immune response (GO:0006955).

### The effect of IDH^mut^ on CpG methylation varies according to cellular differentiation

**Figure 6:**
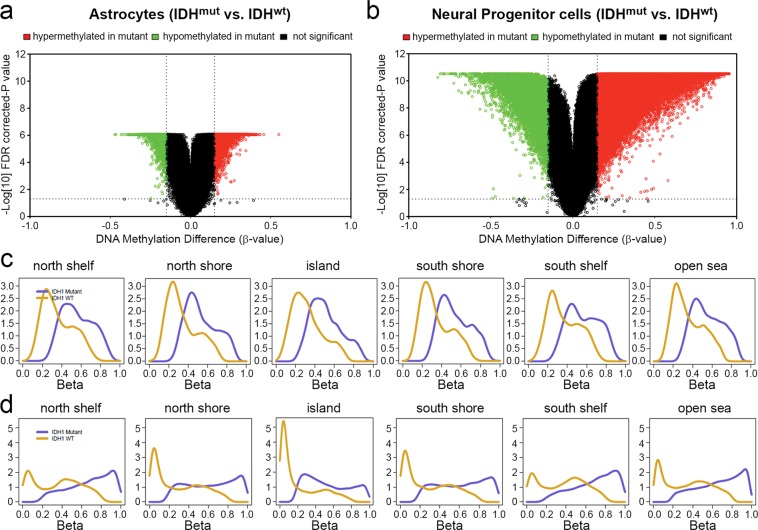
Differences in plasticity of DNA methylation in IDH^mut^ astrocytes and neural progenitor cells. Volcano plots comparing CpG methylation levels of IDH^mut^ versus IDH^wt^ in (**a**) astrocytes and (**b**) neural progenitor cells. Difference of mean methylation (*x*-axis) and significance of the difference (*y*-axis). Each point represents a unique CpG site. Hypermethylation is represented by a delta beta ≥0.15 and FDR-corrected *P* < 0.05 shown in red, and hypomethylation by delta beta ≤−0.15 and FDR-corrected *P* < 0.05. Distribution of mean site-specific CpG methylation across genomic regions for IDH^wt^ and IDH^mut^ (**c**) astrocytes and (**d**) neural progenitor cells.

A causal link between IDH^mut^ and genomic hypermethylation was originally established by inserting IDH^mut^ into IDH^wt^ cells and observing its effects on methylation^5,22^. However, the choice of IDH^wt^ cell models varies greatly among laboratories, even among those focusing on the same kind of IDH^mut^ tumor. For example, in gliomas, some use immortalized differentiated normal human astrocytes (NHAs), whereas others use immature neural progenitor cells (NPCs). To compare the effects of IDH^mut^-induced methylation between different experimental models from the same type of tissue, two publicly available datasets were analyzed: (i) Infinium 450K methylation data from NHAs expressing ectopic R132H IDH1 or IDH1^wt^ for 15 consecutive passages (GSE30339)^5^; (ii) Infinium 450K methylation data from NPCs, also expressing ectopic R132H IDH1 or IDH1^wt^ for 15 consecutive passages (GSE94962)^22^. R132H IDH1 had a far greater effect on the methylation of undifferentiated NPCs than of mature NHAs, resulting in 87,541 versus 15,976 hypermethylated CpG sites, respectively (Fig. 6a,b). Basal methylation levels of IDH^wt^ NPCs compared with IDH^wt^ astrocytes showed extensive hypomethylation, suggesting that they may be more amenable to IDH^mut^ (Supplementary Fig. S6). NPCs with IDH^mut^ versus IDH^wt^ showed a much larger shift towards hypermethylation at the ‘island’, ‘north shore’, and ‘south shore’ genomic regions when compared to NHAs (Fig. 6c,d). Since these studies were analyzed retrospectively and performed using separate experimental conditions, caution must be used when interpreting the results. However, these data may suggest that the effect of IDH^mut^ on the methylome not only depends on cellular lineage, but also on the differentiation state and preexisting DNA methylation of the cell.

## Discussion

The first published case of an IDH^mut^ cancer was in 2006, when a study of over 1,600 breast and colorectal cancers found a single colorectal adenocarcinoma with the R132C variant of IDH1^23^. In 2008–2009, a pair of studies reported a surprisingly high proportion of infiltrative gliomas with IDH^mut^, most commonly R132H IDH1; such tumors tended to be much less aggressive than their IDH^wt^ counterparts^7,24^. Follow-up studies showed that, while IDH^mut^ can rarely be found in a variety of cancers, it is most common in glioma, acute myeloid leukemia, cholangiocarcinoma, melanoma, and cartilaginous tumors^7–11,24^. This was puzzling, insofar as none of the cancers, or the tissues from which they arise, have any obvious connections with each other. Although IDH^mut^ produces D2HG and causes genomic hypermethylation in each type of cancer, only in gliomas does IDH^mut^ show a consistent, powerful association with prognosis (Fig. 1). Guilhamon *et al*. previously reported on shared signaling pathway alterations in IDH^mut^ cancers^25^, yet to the best of our knowledge, no study has focused on what distinguishes IDH^mut^ gliomas from the others. Our TCGA-based data strongly suggests that IDH^mut^ has a unique effect on the methylome and transcriptome of gliomas relative to other IDH^mut^ cancers, and that this could help explain why IDH^mut^ is only a favorable prognostic marker in gliomas.

Assuming that mutations in dividing cells occur in a more or less stochastic fashion, the study of mutation patterns among cancers raises provocative questions regarding the conditions under which specific alterations exert a positive (or negative) evolutionary advantage. In contrast to *TP53*, which is frequently mutated in a wide range of cancers, IDH^mut^ is only enriched in a relatively small number of malignancies, none of which bear any clear resemblance to each other. This would seem to indicate that IDH^mut^, and its D2HG product, are beneficial to cell growth and oncogenesis in only a few settings. Perhaps only a few tissues express wild-type IDH1 and IDH2 in sufficient amounts, or perhaps carbon flux through these enzymes is only strong enough in particular microenvironments, for IDH^mut^ to have a significant effect on metabolism. Wild-type IDH1 and IDH2 normally produce α-ketoglutarate (α-KG) and reduce nicotinamide adenine dinucleotide phosphate (NADPH); the latter then serves as an important antioxidant in the cell^6^. Because IDH^mut^ consumes α-KG and NADPH, some cell types may be better able to compensate for the resultant metabolic deficiencies than others. Since D2HG acts as a competitive inhibitor of certain demethylating dioxygenases that require α-ketoglutarate as a cofactor^6^, the pro-methylating (and pro-oncogenic) effects of IDH^mut^ may depend on the degree to which specific cells express those dioxygenases. And, since a consistent effect of IDH^mut^ is increased genomic methylation, its ability to promote cellular transformation may depend on the patterns of euchromatin and heterochromatin in the cell, which in turn would dictate which regions of the genome are amenable to methylation.

Even among IDH^mut^ cancers, there exists a remarkable preference for certain IDH^mut^ variants according to cancer type. For example, AML has a high proportion of mutations in IDH2, whereas the others are mostly IDH1 (Supplementary Figs S3 and S4). This variant still produces D2HG, but IDH2 is localized to the mitochondrial matrix, while IDH1 is in the cytosol and peroxisomes^6^. IDH^mut^ glioma is distinct in being the only cancer in the entire body to show enrichment for R132H IDH1 (Supplementary Fig. S4). Indeed, R132H IDH1 is so prevalent in gliomas, compared to other variants that are far more common in other IDH^mut^ cancers, it has become standard practice to routinely screen gliomas with an R132H-specific antibody^6^. Yet R132H also appears to be the least efficient producer of D2HG, compared to all other IDH1 and IDH2 variants^26^. This could mean that, in the unique microenvironment of the brain, less D2HG is required for genomic hypermethylation and neoplastic transformation, and that the capacity of glial cells (or glial progenitor cells) to tolerate very high D2HG may be lower than cells elsewhere in the body.

These data also raise the question as to why there is such heterogeneity among CpG methylation sites, particularly in gliomas relative to the other cancers. Even in IDH^mut^ glioma, which shows the greatest extent of CpG hypermethylation compared to its IDH^wt^ counterpart (Fig. 2), not every CpG site is methylated, and not every gene amenable to methylation-induced silencing is suppressed (Supplementary Table S3), or IDH^mut^ tumors would probably never form. To reiterate, the D2HG product of IDH^mut^ only inhibits demethylases, and does not directly cause methylation. In addition to tissue- and cell lineage-specific differences in baseline patterns of euchromatin and heterochromatin, there are other downstream mechanisms that determine which CpG sites are methylated and which ones are not. Such mechanisms include targeting of specific CpG islands, as those regions show the greatest cancer-related variations in IDH^mut^ versus IDH^wt^ methylation (Fig. 3). Prominent among possible mechanisms is cell-specific regulation of DNA methyltransferases, through altered expression, degradation, and complex-mediated targeting of specific CpG sites, which would greatly influence the extent and targets of IDH^mut^-induced methylation^27^. This could help explain some interesting features of IDH^mut^ gliomas. For example, it is well known that *EGFR* mutation and/or amplification, which occurs in nearly 50% of IDH^wt^ gliomas, is nearly mutually exclusive with IDH^mut^ ^28^. Our data indicate that *EGFR* is strongly and uniquely hypermethylated in IDH^mut^ gliomas (Supplementary Spreadsheet) (Supplementary Fig. S5a); such silencing would likely diminish any selection pressure toward activating *EGFR* alterations^29,30^. Conversely, IDH^mut^ gliomas can have *PDGFRA* amplification^31^, and the *PDGFRA* gene is mostly hypomethylated in this subset of gliomas (Supplementary Fig. S5a)^32^. Similarly, *PROM1* is markedly hypermethylated and downregulated only in IDH^mut^ gliomas, not other IDH^mut^ cancers (Supplementary Fig. S5). *PROM1* encodes CD133, a well-known marker of tumor-self-renewal and tumor malignancy^33^. One of the phenotypic hallmarks of high CD133-expressing tumors is the ability to grow as patient-derived xenografts in immunocompromised mice—an ability that IDH^mut^ gliomas, but not other IDH^mut^ cancers, typically lack^6^.

Analysis of tumor-specific patterns in IDH^mut^-associated methylation and mRNA transcription yielded other interesting differences, as well as some profound similarities, among IDH^mut^ malignancies (Figs 4 and 5). All four IDH^mut^ cancers showed relative suppression of genes involved in tissue development (Fig. 5). This has been found to be a consistent effect of IDH^mut^ by multiple laboratories^22,34–37^, and helps explain why IDH^mut^ is associated with neoplasia. While neither *IDH1* nor *IDH2* are classic oncogenes or tumor suppressor genes, and IDH^mut^ is not enough to cause cancer by itself, it may facilitate oncogenesis by extending the window of vulnerability in which additional pro-oncogenic mutations can arise, such as those involving *TP53* in IDH^mut^ astrocytomas^6^. Another common feature of IDH^mut^ cancers is the downregulation of genes associated with immune response (Fig. 5). This is increasingly becoming a recognized feature of IDH^mut^ gliomas^38–41^; our data show that this applies to all IDH^mut^ malignancies, with implications for the efficacy of immune-based therapies in this subset of cancers.

Studies that have demonstrated a direct causal link between IDH^mut^, its D2HG product, and genomic hypermethylation have often done so by inserting one of the IDH^mut^ variants into IDH^wt^ cells, then passaging those cells multiple times and assessing their methylation via the Infinium 450K array. However, the choice of cell model has varied greatly from group to group. Data from two of those models, one expressing R132H IDH1 in differentiated, immortalized human astrocytes^5^, and the other expressing the same mutation in less differentiated neural progenitor cells^22^, for the same number of passages apiece, demonstrate that the ability of R132H IDH1 to promote methylation is much greater in less differentiated cells (Fig. 6). This is consistent with the prevailing thinking, based on patient data, that IDH^mut^ occurs at a very early step in oncogenesis, in cells that have not yet fully differentiated^42^. Whether this window of sensitivity to IDH^mut^ is due to greater amounts of open euchromatin in earlier stages of differentiation, and/or other downstream modulators as discussed above, is not yet clear. But these data do suggest that the choice of cell differentiation state, as well as cell lineage, could greatly impact the results generated in experimental IDH^mut^ research. It may also help explain conflicting results in the literature, including why some investigators have found that IDH^mut^ impairs cancer cell malignancy, whereas others have reported no difference, or even that IDH^mut^ enhances malignant behavior. The same holds for preclinical studies of IDH^mut^ inhibitors, in which some have reported responses to targeted inhibition, whereas others have not.

Finally, these data raise the question as to why certain settings, in which IDH^mut^ showed the greatest amount of hypermethylation, also showed increased hypomethylation relative to matched IDH^wt^ tumors (Figs 2a and 6b). To date, the IDH^mut^ literature has focused on hypermethylation, but these data suggest that there may be another aspect to the effects of IDH^mut^ on the genome, perhaps in the form of increased methylation turnover, or alteration of chromatin structure via IDH^mut^-induced histone methylation, leading to altered accessibility of DNA for methylation.

There are some limitations to this study, including the comparatively fewer numbers of non-glial cancers with IDH^mut^. However, our analyses showed CpG sites and mRNA transcripts that were specifically altered in non-glioma IDH^mut^ cancers, but not in gliomas (Figs 4 and 5). Furthermore, well-described aspects of IDH^mut^ biology, including suppression of differentiation and immunity, were recapitulated in all four IDH^mut^ cancers, thus supporting measured interpretation of intergroup comparisons. The data are also not merely a consequence of IDH^wt^ gliomas being hypomethylated relative to the other IDH^wt^ cancers, because a direct comparison between IDH^mut^ gliomas and other IDH^mut^ malignancies demonstrate that IDH^mut^ gliomas methylate a greater proportion of their genome (Supplementary Tables S1, S2, and Fig. S2). The focus of this project was on TCGA cancers, which does not include chondrosarcomas, another cancer in which IDH^mut^ is frequently seen^11^. Finally, histone methylation data is not present in TCGA, which means that it is not yet clear whether there are also tissue-specific effects of IDH^mut^ on histone modifications.

These data represent a novel comparative analysis of IDH^mut^ cancers, yielding several key insights. While IDH^mut^ does have similar biochemical effects in all tissue types, there is a great deal of heterogeneity regarding the results of that altered biochemistry on the cellular genome and transcriptome. This likely plays a major role in determining whether, and how, IDH^mut^ affects cancer behavior, specifically concerning prognosis and response to IDH^mut^ inhibitors. Furthermore, research involving IDH^mut^ must take into careful consideration the choice of cell model, including the differentiation state of the cell. This study provides a compelling rationale for new lines of investigation into the biology of IDH^mut^, including studies that simultaneously compare several types of cancer, and explore the impact of cellular context.

## Methods

### Analysis of DNA methylation

DNA methylation data from Illumina Human Methylation 450K arrays were downloaded from the Cancer Genome Atlas using the TCGAWorkflow and TCGAbiolinks R packages^43,44^. Legacy data with methylation levels expressed as β-values were downloaded during October 2018^20^. Primary solid tumor data were downloaded for glioma and cholangiocarcinoma. Primary solid tumor and metastatic tumor data were downloaded for melanoma.

### CpG genomic region methylation analysis

CpG sites were mapped to regions (North Shelf, North Shore, Island, South Shore, South Shelf, Open Sea) using the IlluminaHumanMethylation450kanno.ilmn12.hg19 R package.

### Analysis of gene expression

Transcriptome profiles from the Illumina HiSeq platform were downloaded from The Cancer Genome Atlas using the TCGAWorkflow and TCGAbiolinks R packages^43,44^. Harmonized data with gene expression levels quantified as counts were downloaded during October 2018. Data were normalized according to the ‘gcContent’ method^45^. Primary solid tumor data were downloaded for glioma and cholangiocarcinoma. Primary solid tumor and metastatic tumor data were downloaded for melanoma.

### Gene ontology analysis

Gene Ontology (GO) analyses were conducted using differentially expressed genes comparing IDH^mut^ versus IDH^wt^ within each cancer type. The cutoff value for the differentially expressed genes was set *P* < 0.05. The web-based application Metascape (http://www.metascape.org) was used to determine significantly enriched GO biological processes^46^. The Metascape analysis was performed using the default settings.

### Gene set enrichment analysis (GSEA) and network construction

Mean differential gene expression analysis was conducted on IDH^mut^ and IDH^wt^ cancers using the gene set enrichment analysis (GSEA) Desktop v3.0 software (http://www.software.broadinstitute.org/gsea/index.jsp). The number of gene permutations was set to 1,000. The gene-sets were derived from the Broad institute’s Molecular Signatures Database (MSigDB)^47^. Network data visualization and analysis were performed with Cytoscape v3.5.1 software (http://www.cytoscape.org) and with the EnrichmentMap plugin application^48,49^. EnrichmentMap threshold settings: false-discovery rate (FDR) q-value < 0.05; overlap coefficient cutoff = 0.5; *P*-value < 0.05. The functional networks of nodes that represent generic function were determined using the Cytoscape AutoAnnotate application^50^.

### Meta-analysis of astrocyte and neural progenitor cell methylation

For the analysis comparing genome methylation in IDH^mut^ astrocytes and neural progenitor cells data were downloaded from the Gene Expression Omnibus (GEO) (http://www.ncbi.nlm.nih.gov/geo/) using data sets GSE30339 and GSE94962^5,22^. In brief, immortalized human astrocytes were modified to express the IDH1 (R132H) mutation, cultured for 15 passages, and DNA extracted for methylation analysis. Illumina Methylation 450 K data from these data sets were then analyzed using the same approach described in the analysis of DNA methylation methods section.

### Statistical analysis

Differential methylation was evaluated according to Wilcoxon rank sum tests with false discovery rate (FDR) correction. Differential gene expression was evaluated using the edgeR package with FDR correction^51^. Venn diagrams and heatmaps were used to visualize pairwise comparisons of methylation and gene expression.

## Supplementary information

Supplementary Figures

Supplementary Spreadsheet 1

## Data Availability

The results shown here are in part based upon data generated by the TCGA Research Network (http://cancergenome.nih.gov/). Methylation profiles of astrocytes and neural progenitor cells was downloaded from the GEO (http://www.ncbi.nlm.nih.gov/geo/) using data set GSE30339 and GSE94962.
