# Supplementary material for: Methylation and transcription patterns are distinct in IDH mutant gliomas compared to other IDH mutant cancers
Source: Sci Rep. 2019 Jun 20;9:8946. doi: 10.1038/s41598-019-45346-1 (PMC6586617; doi:10.1038/s41598-019-45346-1)
Supplement: Supplementary file 1 — Supplementary Figures [file 41598_2019_45346_MOESM1_ESM.pdf]

Supplemental Table 1: Differential methylation CpG site frequency across IDH^{mut} versus IDH^{wt} cancers.

	Significantly different CpGs (absolute count)	Total CpGs (absolute count)	(% CpGs)
Glioma			
hypermethylated	70,665	365,092	19.2%
hypomethylated	2,928	365,092	0.8%
AML			
hypermethylated	13,128	393,152	3.3%
hypomethylated	125	393,152	0.0%
Cholangio			
hypermethylated	11,763	379,101	3.1%
hypomethylated	926	379,101	0.2%
Melanoma			
hypermethylated	8,663	373,827	2.3%
hypomethylated	34	373,827	0.0%

Cholangio = cholangiocarcinoma

Supplemental Table 2: Differential methylation CpG site frequency comparing IDH^{mut} cancers.

IDH^{mut} vs. IDH^{mut}	affected CpGs (absolute count)	Total CpGs (absolute count)	(% CpGs)
Glioma vs. AML			
hypermethylated	44,880	364,452	12%
hypomethylated	29,046	364,452	8%
Glioma vs. Cholangio			
hypermethylated	43,321	359,851	12%
hypomethylated	31,734	359,851	9%
Glioma vs. Melanoma			
hypermethylated	80,755	353,608	23%
hypomethylated	23,341	353,608	7%

Cholangio = cholangiocarcinoma

Supplemental Table 3: Hypermethylated genes that had differential gene expression

	Number of genes with at least one hypermethylated CpG site	Number of differentially expressed genes	Overlap (%)
Glioma	12625	4691	2611 (37)
AML	5356	282	76 (5)
Melanoma	3488	758	143 (22)
Cholangio	4789	246	89 (5)

Cholangio = cholangiocarcinoma; AML = acute myeloid leukemia

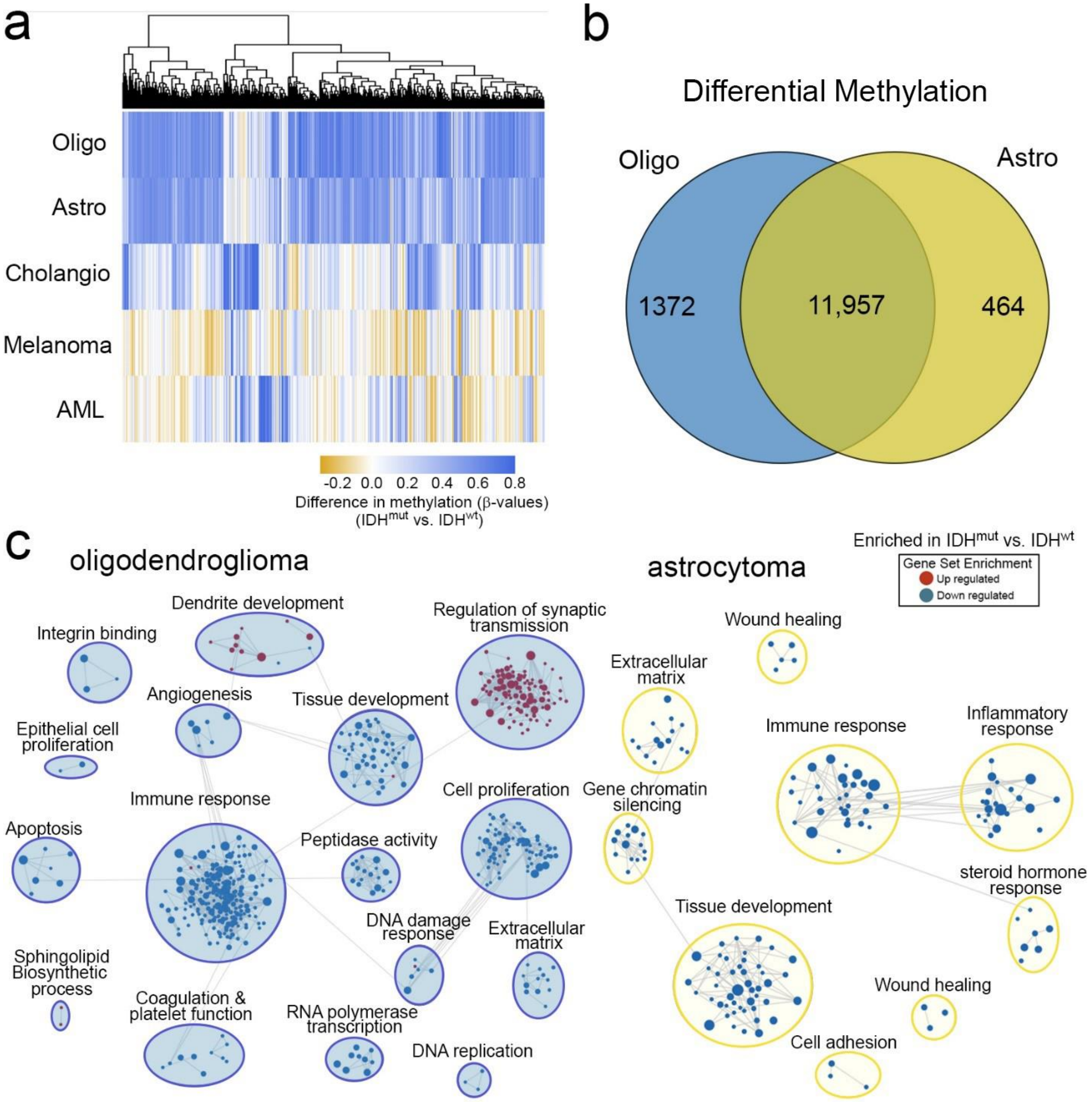

Supplementary Figure 1. Differential methylation and transcription signatures between IDH^{mut} oligodendroglioma or astrocytoma and IDH^{wt} gliomas. (**a**) Methylation heatmap of differences in CpG site beta values between IDH^{mut} and IDH^{wt} tumors for all genes demonstrating statistically significant hypermethylation. (**b**) Venn diagram representing the overlap of hypermethylated genes of IDH^{mut} oligodendrogliomas and astrocytomas compared with IDH^{wt} gliomas. (**c**) Gene Set Enrichment Analysis (GSEA) showing gene sets in an interaction network for

oligodendroglioma (blue) and astrocytoma (yellow). Each circular node represents enriched gene sets. Blue nodes represent negative enrichment and red nodes represent positive enrichment. Edges (grey connection lines) represent overlap between gene sets with line thickness correlating to the degree of overlap. Networks of nodes that reflect generic function were circled in blue (oligodendroglioma) or yellow (astrocytoma) and assigned group labels.

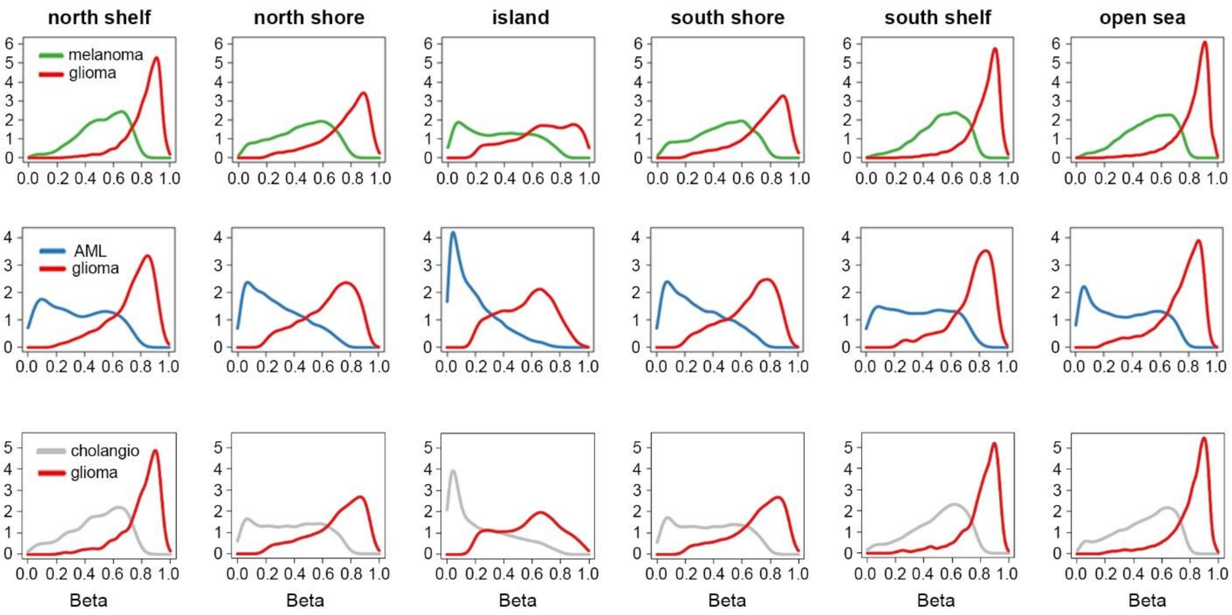

Supplemental Figure 2. Distribution of mean site-specific CpG methylation levels by genomic region for CpG sites identified as hypermethylated in IDH^{mut} gliomas versus other IDH^{mut} cancer types. The density of probes at each CpG site (*y*-axis) and methylation β-values (*x*-axis). Islands are genomic areas with relatively high CpG content flanked by shores (up to 2 kilobases) and shelves (2-4 kilobases from island). North shore and shelf represent 5' end, and south shore and shelf represent 3' end. The open sea represents the rest of the genome. Red lines represent CpG methylation in glioma tumors, green lines melanoma, blue lines AML, and purple lines cholangiocarcinoma.

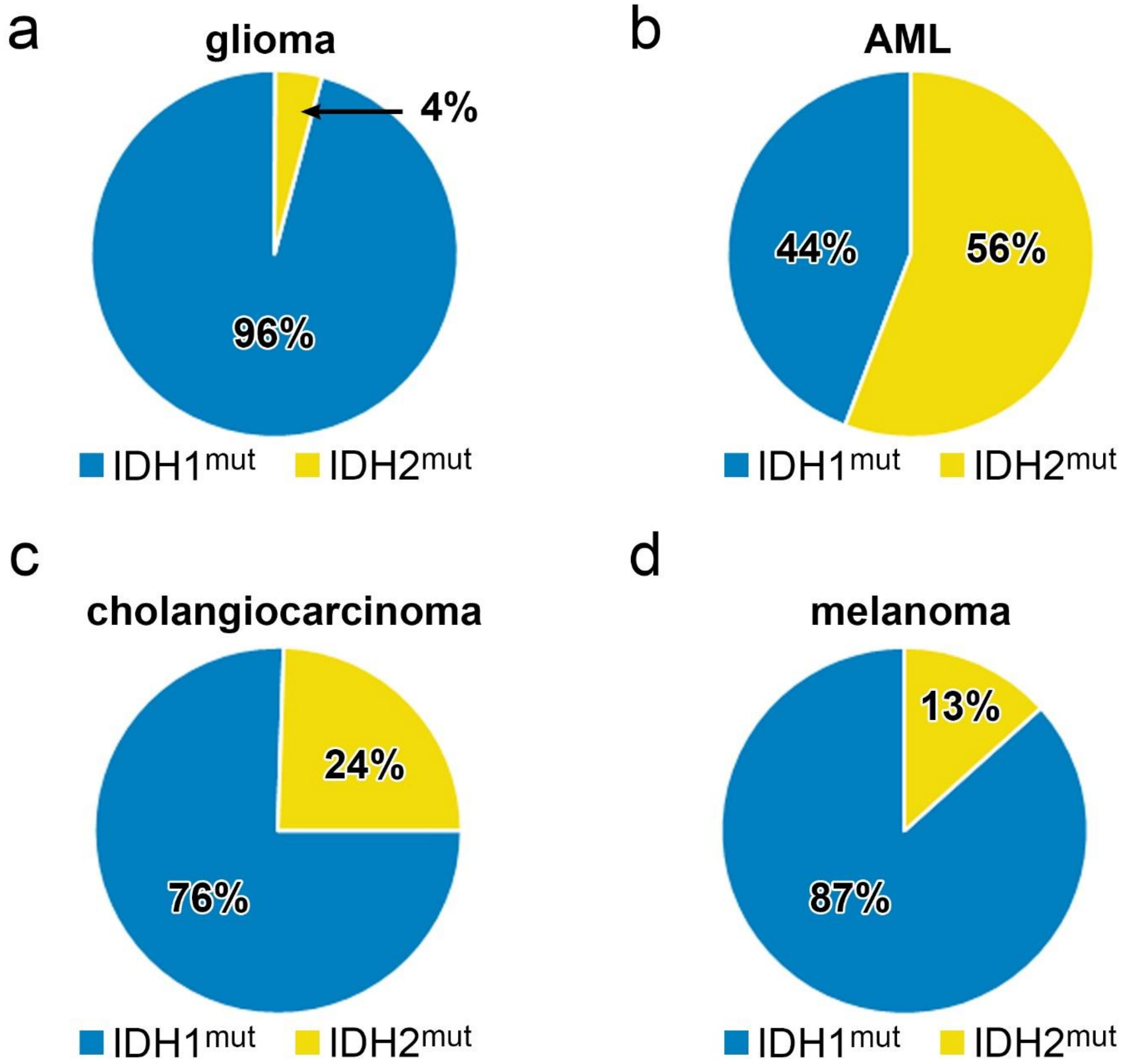

Supplemental Figure 3: Meta-analysis of IDH1 and IDH2 mutation frequency associated with each cancer. Pie charts for percent IDH1 and IDH2 mutation frequency for (**a**) glioma ($IDH1^{mut}$ N=1375; $IDH2^{mut}$ N=59; total=1434) [1], (**b**) AML ($IDH1^{mut}$ N=228; $IDH2^{mut}$ N=288; total=516) [2-11], (**c**) cholangiocarcinoma ($IDH1^{mut}$ N=74; $IDH2^{mut}$ N=24; total=98) [12-17], and (**d**) melanoma ($IDH1^{mut}$ N=13; $IDH2^{mut}$ N=2; total=15) [18-20].

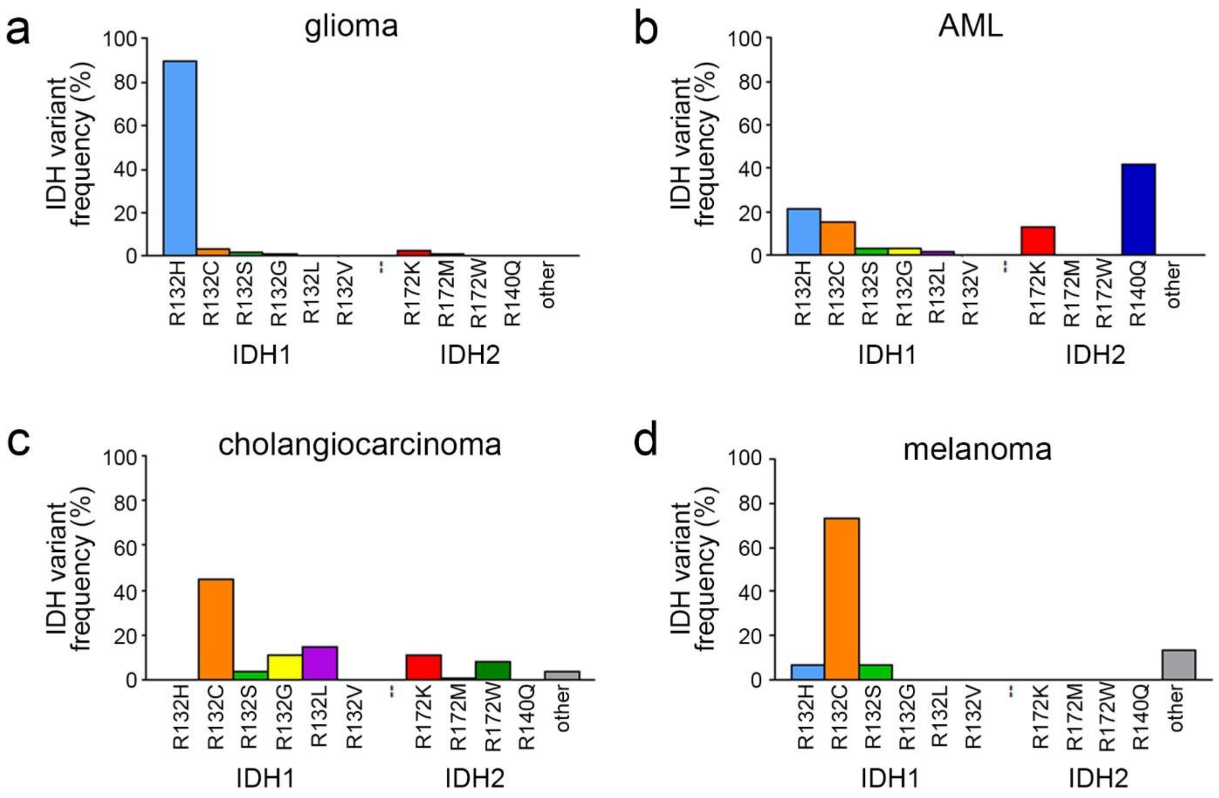

Supplemental Figure 4: Meta-analysis of IDH mutation type frequency associated with each cancer. IDH mutation variant frequency for (**a**) glioma (IDH1^{mut} N=1375; IDH2^{mut} N=59; total=1434) [1], (**b**) AML (IDH1^{mut} N=228; IDH2^{mut} N=288; total=516) [2-11], (**c**) cholangiocarcinoma (IDH1^{mut} N=74; IDH2^{mut} N=24; total=98) [12-17], and (**d**) melanoma (IDH1^{mut} N=13; IDH2^{mut} N=2; total=15) [18-20].

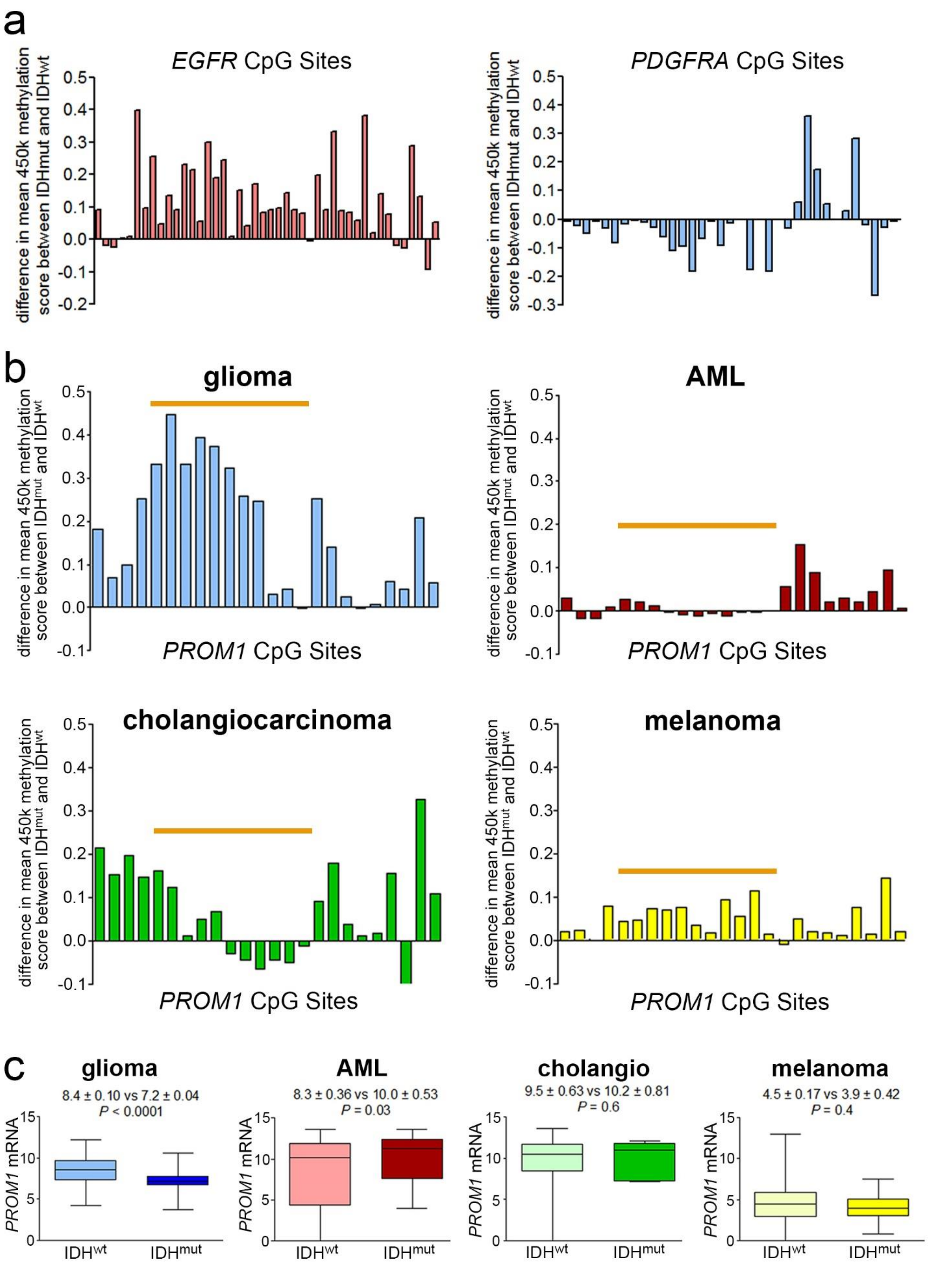

a
EGFR CpG Sites
PDGFRA CpG Sites
difference in mean 450k methylation score between IDHmut and IDHwt
0.5
0.4
0.3
0.2
0.1
0.0
-0.1
-0.2
-0.3
b
glioma
AML
cholangiocarcinoma
melanoma
difference in mean 450k methylation score between IDHmut and IDHwt
PROM1 CpG Sites
c
glioma
8.4 ± 0.10 vs 7.2 ± 0.04
P < 0.0001
AML
8.3 ± 0.36 vs 10.0 ± 0.53
P = 0.03
cholangio
9.5 ± 0.63 vs 10.2 ± 0.81
P = 0.6
melanoma
4.5 ± 0.17 vs 3.9 ± 0.42
P = 0.4
PROM1 mRNA
15
10
5
0
IDHwt
IDHmut

Supplemental Figure 5: Gene methylation and expression in IDH^{mut} cancers. (**a**) Mean increase in methylation beta values at each CpG site associated with *EGFR* or *PDGFRA*, in IDH^{mut} versus IDH^{wt} gliomas. (**c**) Mean increase in methylation beta values at each CpG site associated with *PROM1*, in IDH^{mut} versus IDH^{wt} cancers (blue = glioma; red = AML; yellow = melanoma; green = cholangiocarcinoma). The orange bars above bar graphs indicates CpG sites that reside within 'islands'. (**b**) *PROM1* mRNA (Log2) expression in IDH^{wt} and IDH^{mut} cancers. The box and whisker plots of gene expression show medium, interquartile range, and minimum and maximum error.

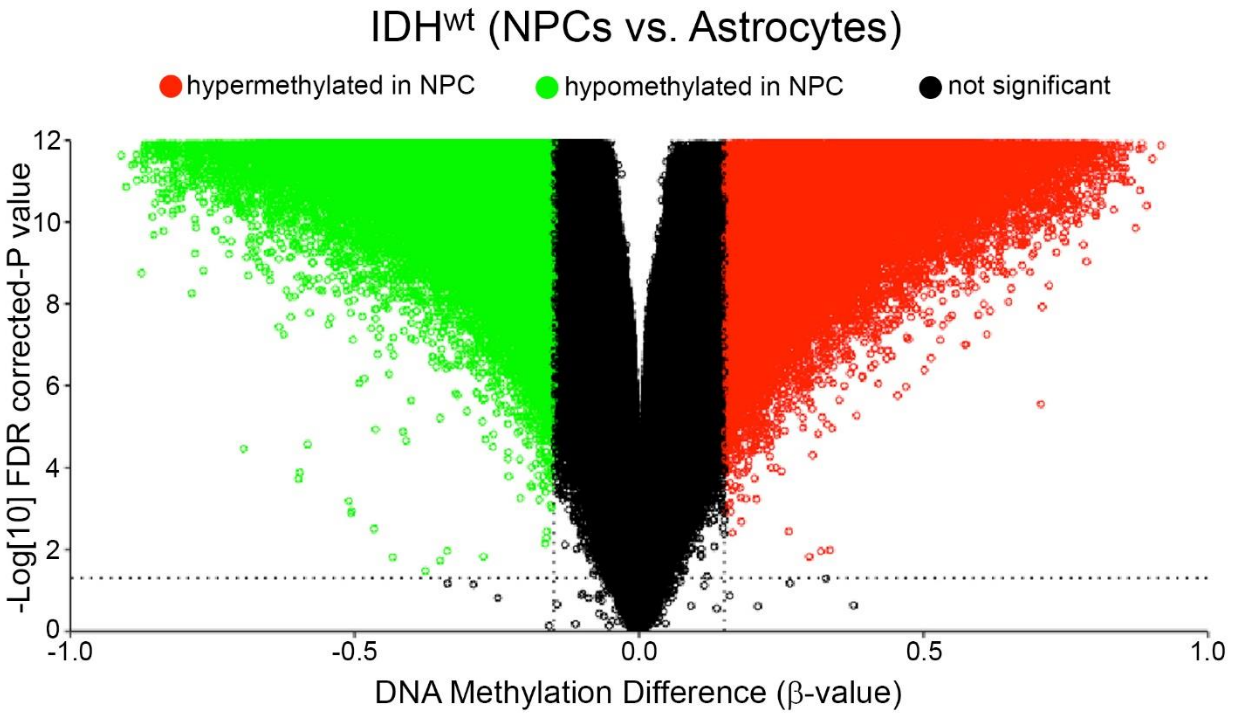

Supplemental Figure 6: Differences in DNA methylation between IDH^{wt} astrocytes and IDH^{wt} neural progenitor cells. Difference of methylation (*x*-axis) and significnace of the difference (*y*-axis). Each point represents a unique CpG site. Hypermethylation is represented by a delta beta ≥ 0.15 and FDR-corrected *P*>0.05 shown in red, and hypomethylation by delta beta ≤ -0.15 and FDR-corrected *P*>0.05.

SUPPLEMENTAL REFERENCES

1 Wang, P. *et al.* Mutations in isocitrate dehydrogenase 1 and 2 occur frequently in intrahepatic cholangiocarcinomas and share hypermethylation targets with glioblastomas. *Oncogene* **32**, 3091-3100, doi:10.1038/onc.2012.315 (2013).

2 Abbas, S. *et al.* Acquired mutations in the genes encoding IDH1 and IDH2 both are recurrent aberrations in acute myeloid leukemia: prevalence and prognostic value. *Blood* **116**, 2122-2126, doi:10.1182/blood-2009-11-250878 (2010).

3 Boissel, N. *et al.* Prognostic impact of isocitrate dehydrogenase enzyme isoforms 1 and 2 mutations in acute myeloid leukemia: a study by the Acute Leukemia French Association group. *Journal of clinical oncology : official journal of the American Society of Clinical Oncology* **28**, 3717-3723, doi:10.1200/jco.2010.28.2285 (2010).

4 Chotirat, S., Thongnoppakhun, W., Promsuwicha, O., Boonthimat, C. & Auewarakul, C. U. Molecular alterations of isocitrate dehydrogenase 1 and 2 (IDH1 and IDH2) metabolic genes and additional genetic mutations in newly diagnosed acute myeloid leukemia patients. *Journal of hematology & oncology* **5**, 5, doi:10.1186/1756-8722-5-5 (2012).

5 Chou, W. C. *et al.* Distinct clinical and biologic characteristics in adult acute myeloid leukemia bearing the isocitrate dehydrogenase 1 mutation. *Blood* **115**, 2749-2754, doi:10.1182/blood-2009-11-253070 (2010).

6 DiNardo, C. D. *et al.* Serum 2-hydroxyglutarate levels predict isocitrate dehydrogenase mutations and clinical outcome in acute myeloid leukemia. *Blood* **121**, 4917-4924, doi:10.1182/blood-2013-03-493197 (2013).

7 Koszarska, M. *et al.* Type and location of isocitrate dehydrogenase mutations influence clinical characteristics and disease outcome of acute myeloid leukemia. *Leukemia & lymphoma* **54**, 1028-1035, doi:10.3109/10428194.2012.736981 (2013).

8 Marcucci, G. *et al.* IDH1 and IDH2 gene mutations identify novel molecular subsets within de novo cytogenetically normal acute myeloid leukemia: a Cancer and Leukemia Group B study. *Journal of clinical oncology : official journal of the American Society of Clinical Oncology* **28**, 2348-2355, doi:10.1200/jco.2009.27.3730 (2010).

9 Mardis, E. R. *et al.* Recurring mutations found by sequencing an acute myeloid leukemia genome. *N Engl J Med* **361**, 1058-1066, doi:10.1056/NEJMoa0903840 (2009).

10 Paschka, P. *et al.* IDH1 and IDH2 mutations are frequent genetic alterations in acute myeloid leukemia and confer adverse prognosis in cytogenetically normal acute myeloid leukemia with NPM1 mutation without FLT3 internal tandem duplication. *Journal of clinical oncology : official journal of the American Society of Clinical Oncology* **28**, 3636-3643, doi:10.1200/jco.2010.28.3762 (2010).

11 Thol, F. *et al.* IDH1 mutations in patients with myelodysplastic syndromes are associated with an unfavorable prognosis. *Haematologica* **95**, 1668-1674, doi:10.3324/haematol.2010.025494 (2010).

12 Goyal, L. *et al.* Prognosis and Clinicopathologic Features of Patients With Advanced Stage Isocitrate Dehydrogenase (IDH) Mutant and IDH Wild-Type Intrahepatic Cholangiocarcinoma. *The oncologist* **20**, 1019-1027, doi:10.1634/theoncologist.2015-0210 (2015).

13 Jiao, Y. *et al.* Exome sequencing identifies frequent inactivating mutations in BAP1, ARID1A and PBRM1 in intrahepatic cholangiocarcinomas. *Nature genetics* **45**, 1470-1473, doi:10.1038/ng.2813 (2013).

14 Kipp, B. R. *et al.* Isocitrate dehydrogenase 1 and 2 mutations in cholangiocarcinoma. *Human pathology* **43**, 1552-1558, doi:10.1016/j.humpath.2011.12.007 (2012).

15 Borger, D. R. *et al.* Frequent mutation of isocitrate dehydrogenase (IDH)1 and IDH2 in cholangiocarcinoma identified through broad-based tumor genotyping. *The oncologist* **17**, 72-79, doi:10.1634/theoncologist.2011-0386 (2012).

16 Wang, J. *et al.* IDH1 mutation correlates with a beneficial prognosis and suppresses tumor growth in IHCC. *The Journal of surgical research* **231**, 116-125, doi:10.1016/j.jss.2018.04.056 (2018).
17 Zhu, A. X. *et al.* Genomic profiling of intrahepatic cholangiocarcinoma: refining prognosis and identifying therapeutic targets. *Annals of surgical oncology* **21**, 3827-3834, doi:10.1245/s10434-014-3828-x (2014).
18 Linos, K. & Tafe, L. J. Isocitrate dehydrogenase 1 mutations in melanoma frequently co-occur with NRAS mutations. *Histopathology*, doi:10.1111/his.13707 (2018).
19 Lopez, G. Y. *et al.* IDH1(R132) mutation identified in one human melanoma metastasis, but not correlated with metastases to the brain. *Biochemical and biophysical research communications* **398**, 585-587, doi:10.1016/j.bbrc.2010.06.125 (2010).
20 Shibata, T., Kokubu, A., Miyamoto, M., Sasajima, Y. & Yamazaki, N. Mutant IDH1 confers an in vivo growth in a melanoma cell line with BRAF mutation. *The American journal of pathology* **178**, 1395-1402, doi:10.1016/j.ajpath.2010.12.011 (2011).
